# Differences in Intrinsic Brain Abnormalities Between Patients With Left- and Right-Sided Long-Term Hearing Impairment

**DOI:** 10.3389/fnins.2019.00206

**Published:** 2019-03-12

**Authors:** Xiaoxiao Xie, Yongbo Liu, Xiaowei Han, Pei Liu, Hui Qiu, Junfeng Li, Huachen Yu

**Affiliations:** ^1^Department of Radiology, The Second Affiliated Hospital and Yuying Children’s Hospital of Wenzhou Medical University, Wenzhou, China; ^2^Department of Radiology, Shanxi Lu’an General Hospital, Changzhi, China; ^3^Graduate School, Chinese Academy of Medical Sciences, Peking Union Medical College, Beijing, China; ^4^Department of Radiology, Heping Hospital of Changzhi Medical College, Changzhi, China; ^5^Graduate School, Beijing University of Chinese Medicine, Beijing, China; ^6^Graduate School, Changzhi Medical College, Changzhi, China; ^7^Department of Orthopedics, The Second Affiliated Hospital and Yuying Children’s Hospital of Wenzhou Medical University, Wenzhou, China

**Keywords:** unilateral hearing impairment, functional magnetic resonance imaging, fractional amplitude of low frequency fluctuation, functional connectivity, brain network

## Abstract

Unilateral hearing impairment is characterized by asymmetric hearing input, which causes bilateral unbalanced auditory afferents and tinnitus of varying degrees. Long-term hearing imbalance can cause functional reorganization in the brain. However, differences between intrinsic functional changes in the brains of patients with left- and those with right-sided long-term hearing impairments are incompletely understood. This study included 67 patients with unilateral hearing impairments (left-sided, 33 patients; right-sided, 34 patients) and 32 healthy controls. All study participants underwent blood oxygenation level dependent resting-state functional magnetic resonance imaging and T1-weighted imaging with three-dimensional fast spoiled gradient-echo sequences. After data preprocessing, fractional amplitude of low frequency (fALFF) and functional connectivity (FC) analyses were used to evaluate differences between patients and healthy controls. When compared with the right-sided hearing impairment group, the left-sided hearing impairment group showed significantly higher fALFF values in the left superior parietal gyrus, right inferior parietal lobule, and right superior frontal gyrus, whereas it showed significantly lower fALFF values in the left Heschl’s gyrus, right supramarginal gyrus, and left superior frontal gyrus. In the left-sided hearing impairment group, paired brain regions with enhanced FC were the left Heschl’s gyrus and right supramarginal gyrus, left Heschl’s gyrus and left superior parietal gyrus, left superior parietal gyrus and right inferior parietal lobule, right inferior parietal lobule and right superior frontal gyrus, and left and right superior frontal gyri. In the left-sided hearing impairment group, the FC of the paired brain regions correlated negatively with the duration and pure tone audiometry were in the left Heschl’s gyrus and right supramarginal gyrus. In the right-sided hearing impairment group, the FC of the paired brain regions correlated negatively with the duration was in the left Heschl’s gyrus and superior parietal gyrus, and with pure tone audiometry was right inferior parietal lobule and superior frontal gyrus. The intrinsic reintegration mechanisms of the brain appeared to differ between patients with left-sided hearing impairment and those with right-sided hearing impairment, and the severity of hearing impairment was associated with differences in functional integration in certain brain regions.

## Introduction

Hearing is an important advanced function of the human brain. The mechanisms underlying hearing and hearing disorders are very complicated (Paul et al., 2017; Ungar et al., 2018). Hearing impairments can cause debilitating problems, such as poor voice location and difficulties with orientation and listening (Chen and Young, 2016; Shah et al., 2018). Unilateral hearing impairments are characterized by asymmetric auditory transmissions accompanied by tinnitus of varying degrees. This causes bilateral imbalance in auditory afferents, importantly, long-term hearing imbalance can trigger cortical reorganization of the brain (Kim et al., 2018).

The incidence of unilateral hearing impairment increases with age. Long-term unilateral hearing impairment can lead to hearing problems, as well as behavioral and psychological deficits (e.g., dysphoria and restlessness) (Paul et al., 2017). Moreover, long-term unilateral hearing impairment can lead to abnormal changes in brain function, which can cause a variety of abnormalities in brain networks involving sensory, conductive, and cognitive functions (Shah et al., 2018). Understanding these abnormal changes in brain function and related functional integration in patients with long-term unilateral hearing impairment could help to elucidate the neurological mechanisms by which impairment of brain functions may occur.

Awareness has increased over the past decade regarding how the brain remodels itself in response to hearing impairment and the mechanisms underlying the processing and integration of auditory information; this increasing awareness is due to the development and advancement of neuroimaging technologies in auditory research, such as magnetoencephalography and functional magnetic resonance imaging (fMRI) (Burton et al., 2012; Hughes et al., 2018). The complex processes of auditory information recognition and functional coding undergo restructuring in response to hearing impairment (Andoh et al., 2015). One study has suggested the presence of a frequency-sensitive coding structure in the auditory cortex of the human brain (Chang et al., 2016). The dominance of the contralateral hemisphere disappears when a unilateral auditory stimulus is presented (Andoh and Zatorre, 2011). Previous studies have shown that the pattern of auditory conduction on the side of hearing impairment can significantly affect the lateralization pattern of the auditory cortex (Chen and Young, 2016). In unilateral hearing impairment, uniaural sound conduction is present, which produces asymmetric activation patterns of unilateral neurons in the auditory network of the brain, thereby leading to dominant contralateral functional activity of the auditory projection. In addition, in long-term unilateral hearing impairment, activation of the auditory cortex tends to shift to a more symmetrical and synchronous mode of auditory conduction. Thus far, some studies in neuroimaging have confirmed that a subset of functional networks of the brain associated with integration of auditory networks may change in patients with unilateral hearing impairment. The function and structure of the auditory systems are reorganized in patients with unilateral hearing impairment (Bertolero et al., 2015). Recent studies have shown that the remodeling of hearing function occurs in patients with unilateral hearing impairment (Zhang et al., 2017a); moreover, this correlates with the patients’ mental states (Kompus et al., 2012; Firszt et al., 2017). However, changes in functional activity in different regions of the brain, as well as abnormal functional connectivity (FC) patterns that could develop due to unilateral hearing impairment, remain unexplored.

In resting-state fMRI (R-fMRI), the amplitude of low frequency fluctuation (ALFF) and fractional amplitude of low frequency fluctuation (fALFF) reflect spontaneous activity in the brain (Jing et al., 2013). At low frequencies, fALFF is more sensitive and specific than ALFF, and can more accurately reflect the strength of functional activity in the brain (Zou et al., 2008). FC, defined as the correlation between a temporal series and the corresponding functionally activated events, is another method of quantification (van de Ven et al., 2004). Both fALFF and FC have been used as reliable and sensitive indicators in healthy subjects and in patients with various neurological and psychiatric diseases, such as Alzheimer’s disease and depression. Additionally, some neuroimaging studies have used these methods and have reported that patients with hearing impairment exhibit abnormal intrinsic functional activities in the brain, which are involved in the auditory network (Leaver and Rauschecker, 2016). However, these quantitative methods have not been used in patients with unilateral hearing impairment, and the relationship between quantified spontaneous neurological activity, as determined by these two methods, and clinical measurements of unilateral hearing impairment has not been studied. Furthermore, regions of functional activity in patients with unilateral hearing impairment, as well as subsequent changes in functional networks that are caused by unilateral hearing impairment, remain unclear. Finally, the correlation between these changes and the degree of hearing impairment is unknown.

The purpose of our study was to explore differences in the intrinsic functional changes in the brain between patients with left- and right-sided hearing impairment using fALFF and FC. In this study, we hypothesized that patients with long-term unilateral hearing impairment would exhibit abnormal intrinsic functional activity in relevant areas of the brain and reconstruction of related functional networks. We also hypothesized that this would partially explain differences between patients and healthy controls. We tested this hypothesis using R-fMRI with fALFF and FC analysis. Furthermore, we analyzed the correlation between altered FC and the duration of illness and pure tone audiometry (PTA) scores in patients with left- and right-sided hearing impairments.

## Materials and Methods

### Participants

This study included 67 patients with unilateral hearing impairments caused by idiopathic hearing loss. All patients were right-handed and aged between 28 and 54 years. Left-sided hearing impairment was present in 33 patients (19 men; 14 women), and right-sided hearing impairment was present in 34 patients (19 men; 15 women). Thirty-two healthy, right-handed controls, aged between 24 and 56 years, were also recruited (18 men; 14 women). Patients were excluded for the following reasons: (1) congenital brain dysplasia or accidental brain trauma with structural deformation of the brain or loss of consciousness; (2) addiction or dependence on special substances (history of drug use, analgesic drug intake or alcohol abuse); (3) other neurological or psychiatric diseases, or somatic diseases that may lead to central nervous system dysfunction; (4) active breast-feeding or pregnancy; and (5) refusal to undergo magnetic resonance scanning. A previous study showed that cortical remodeling occurs in patients who have had unilateral hearing impairment for more than 2 years (Schmithorst et al., 2014). Hence, all patients included in this study had been diagnosed with unilateral long-term hearing loss for at least 2.5 years by two senior otologists. The degree of hearing was evaluated using mean PTA thresholds, which were measured and calculated using the same audiometer at thresholds of 500, 1000, 2000, and 4000 Hz to reflect the listening levels of the participants. The affected ears of the patients had moderate to severe hearing impairments (PTA score > 55 dB). The unaffected ears of the patients and the healthy controls had normal hearing (PTA score ≤ 25 dB). The study was approved by the local ethics committee of our hospital, and written informed consent was obtained from each participant.

### Data Acquisition

The participants underwent MRI using a 3.0T MR scanner (General Electric, Discovery MR750, Milwaukee, WI, United States) with a matched eight-channel phased array head coil, and parallel imaging of the brain was employed. The scan protocol included the following: (1) scout images; (2) T2-weighted images; (3) resting-state blood oxygenation level dependent fMRI with single-shot gradient recalled echo-planar echo imaging sequences (GRE-EPI); and (4) three-dimensional T1-weighted images with three-dimensional fast spoiled gradient-echo sequences (3D FSPGR). The parameters of the GRE-EPI included slice thickness of 3.5 mm, slice spacing of 0.7 mm, repetition time of 2000 ms, echo time of 30 ms, flip angle of 90°, matrix size of 64 × 64, field of view of 240 × 240 mm^2^, and number of excitations of 1, 34 slices, and 240 time points. The parameters of the 3D FSPGR included slice thickness of 1.0 mm, repetition time of 6.7 ms, echo time of Min Full, acquisition matrix of 256 × 256, field of view of 256 × 256 mm^2^, and number of excitations of 1. Participants were instructed to rest in the supine position with their eyes closed and to breathe calmly. Their heads were fixed to minimize movement, and they were provided with soft earplugs, and electrostatic headphones for both ears. They were also instructed to remain awake and not to think of anything specific.

### Data Processing and Analysis

#### Data Pre-processing

SPM12^1^ was used for data processing and analysis (Eickhoff et al., 2005). The preprocessing procedure included the following steps: (1) The data format was converted from DICOM to NIFTI, and the first five time points were removed. (2) The slice-timing was corrected. (3) The head motion was corrected in all subjects (the standard was less than 2.5 mm or 2.5°), and three translation and three rotation parameters were obtained using the head motion. (4) Spatial normalization was performed. Functional images were co-registered with corresponding T1 structural images that were segmented and warped into the anatomical Montreal Neurological Institute template; they were then transformed at a resolution of 3-mm isotropic resolution into the standard Montreal Neurological Institute space. (5) Smoothing was performed with a full width at half maximum of 4. (6) The linear drift was removed. (7) Covariate regression was performed, and six head-motion parameters, white matter, cerebrospinal fluid, and global signals were removed by applying linear regression. (8) Band-pass filtering was performed (frequencies were set at 0.01 to 0.08 Hz) to remove the effects of low-frequency drifts and high-frequency noise after fALFF calculation.

#### fALFF and FC Analysis

Fractional amplitude of low frequency fluctuation values from each voxel were obtained from preprocessed data. Differences between the left- and right-sided hearing impairment groups and the healthy controls were analyzed and compared to identify regions between the groups. These coordinates were used as the centers of 4-mm-sized spherical seed brain regions of interest (ROIs) that were created using the wfu_pickatlas tool in SPM12. First, FC analysis was performed by calculating the time serial correlation coefficients between each ROI and all the other non-ROI voxels (voxel-wise) in each participant. The *z*-values were obtained for further statistical analysis from these correlation coefficients using Fisher’s z translation. Intergroup mean comparisons of overall FC of each brain region were conducted between left- and right-sided hearing impairment groups. In addition, the FC between two ROIs (paired ROIs) was calculated, and intergroup multiple comparisons were analyzed.

#### Statistical Analysis

One-way analysis of variance was used to compare age and level of education among the three groups. Two-sample *t*-tests were used to compare the duration of illness and PTA scores between the left- and right-sided hearing impairment groups. The chi-square test was applied to compare sex distributions. Demographic and clinical data were analyzed using SPSS (version 23.0, SPSS, Inc., Chicago, IL, United States).

One-way analysis of covariance was performed to examine group differences in fALFF values. Age, sex, and level of education were incorporated as covariates. *Post hoc* tests were conducted to identify variations between each pair of groups. Intergroup comparisons of the overall FC of each brain region were conducted by independent two-sample *t*-tests. Multiple comparisons of FC between each pair of ROIs were performed, and false discovery rate correction was used for multiple comparison correction in the resulting data with a threshold of 0.05 (Song et al., 2017). The *z*-values of abnormal FC were extracted and used for Pearson correlation analysis with duration of illness and PTA scores of the patients. The differences were considered statistically significant if the value of *P* was less than 0.05.

## Results

### Clinical Data

There were no significant differences in sex, age, or level of education between patients with hearing impairment and healthy controls (*P* > 0.05). The duration of illness was comparable (*P* > 0.05) between patients with left-sided hearing impairment (mean ± standard deviation: 38.50 ± 12.57 months) and those with right-sided hearing impairment (mean ± standard deviation: 37.50 ± 11.72 months). There were no significant differences in the PTA of the affected ear between the left-sided hearing impairment group (mean ± standard deviation: 76.20 ± 24.28 dB) and right-sided hearing impairment group (mean ± standard deviation: 75.60 ± 23.11 dB) (*P* > 0.05; Table 1).

**Table 1:** Demographic and clinical data of unilateral hearing impairment and healthy control patients.

Protocols	Patients		HCs (32)	*P*-value
			
	Left (33)	Right (34)		
Gender (M/F)	19/14	19/15	18/14	0.694^a^
Age (years)	46.15 ± 11.32	45.48 ± 10.67	44.45 ± 10.39	0.125^b^
Education (years)	10.5 ± 1.36	11.20 ± 3.27	11.05 ± 1.77	0.351^b^
Duration (months)	38.50 ± 12.57	37.50 ± 11.72	/	0.362^c^
PTA of affected	76.2 ± 24.28	75.6 ± 23.11	/	0.434^c^
ear (dB HL)


*HCs, healthy control patients; PTA, pure tone audiometry; ^*a*^*P*-value was obtained using a Pearson Chi-square test (two-tailed) among three groups; ^*b*^*P*-value was obtained using one-way ANOVA (two-tailed) among three groups; ^*c*^*P*-value was obtained using the independent two sample *t*-test (two-tailed).*

### Difference in fALFF Between Left- and Right-Sided Hearing Impairment Groups

The fALFF values in the left triangular portion of the inferior frontal gyrus, left middle temporal gyrus, left calcarine, left and right thalami, right middle occipital gyrus, and right superior frontal gyrus were significantly higher in the left-sided hearing impairment group than in the healthy control group (Table 2).

**Table 2:** Brain regions showing group fALFF differences with *post hoc* tests analysis.

Brain regions	BA	Peak MNI Coordinates (mm) (*x, y, z*)	*t*-value
Left vs. right groups			
HG.L	38	(-55, -12, 11)	-2.25
SMG.R	48	(60, -39, 36)	-2.42
SPG.L	7	(-30, -57, 67)	2.48
IPL.R	40	(49, -47, 51)	2.72
SFG.L	6	(-13, 14, 52)	-2.67
SFG.R	8	(23, 13, 61)	2.39
Left vs. healthy controls			
IFGtri.L	45	(-52, 30, 5)	2.58
MTG.L	21	(-60, -53, 7)	2.78
Calcarine.L	17	(-4, -93,5)	2.65
Thalamus.L	/	(-8, -21,8)	2.39
Thalamus.R	/	(8, -23,12)	2.64
MOG.R	19	(32, -85,20)	2.59
SFG.R	6	(23,1,63)	2.71
Right vs. healthy controls			
ITG.R	20	(55, -14, -27)	2.31
PHG.R	20	(35, -18, -20)	2.36
Thalamus.L	/	(-5, -20,2)	2.24
Thalamus.R	/	(5, -17,20)	2.26
IFGtri.L	45	(-54,24,13)	3.26
Caudate.L	25	(-6, 19, -4)	2.43
IPL.L	40	(-35, -42, 43)	2.57


*BA, Brodmann area; MNI, Montreal Neurologic Institute; HG.L, left Heschl’s gyrus; SMG.R, right supramarginal gyrus; SPG.L, left superior parietal gyrus; IPL.R, right inferior parietal lobule; SFG.L, left superior frontal gyrus; SFG.R, right superior frontal gyrus; PreCG.L, left precentral gyrus; IFGtri.L, left triangular part of inferior frontal gyrus; MTG.L, left Middle temporal gyrus; Calcarine.L, left calcarine; Thalamus.L, left thalamus; Thalamus.R, right thalamus; MOG.R, right middle occipital gyrus; ITG.R, right inferior temporal gyrus; PHG.R, right parahippocampal gyrus; Caudate.L, left caudate.*

The fALFF values in the right inferior temporal gyrus, right parahippocampal gyrus, left and right thalami, left triangular portion of the inferior frontal gyrus, left caudate, and left inferior parietal lobule were significantly higher in the right-sided hearing impairment group than in the healthy control group (Table 2).

**FIGURE 1:**
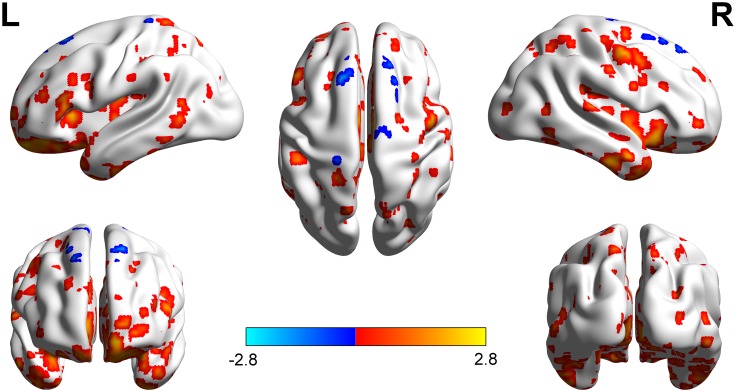
Brain regions showed fALFF differences between left and right hearing impairment groups. The different regions with higher fALFF were observed in the left superior parietal gyrus, right inferior parietal Lobule, and right superior frontal gyrus. The regions with lower fALFF were observed mainly in the left Heschl’s gyrus, right supramarginal gyrus, and left superior frontal gyrus.

Compared with the right-sided hearing impairment group, the left-sided hearing impairment group showed significantly higher fALFF values in the left superior parietal gyrus, right inferior parietal lobule, and right superior frontal gyrus; moreover, the left-sided hearing impairment group showed significantly lower fALFF values in the left Heschl’s gyrus, right supramarginal gyrus, and left superior frontal gyrus (Figure 1 and Table 2).

### Intergroup Comparison of Overall FC

By comparing left- and right-sided hearing impairment groups, we identified six brain regions that showed different fALFF values. However, the mean overall connectivity in these brain regions did not differ between the two groups (*P* > 0.05; Table 3).

**Table 3:** Comparison of the means of the overall inter-regional connectivity between left and right hearing impairment patient groups (mean *z*-values, mean ± SD).

Brain regions	HG.L	SMG.R	SPG.L	IPL.R	SFG.L	SFG.R
Left hearing impairment	1.53 ± 0.32	1.55 ± 0.23	1.59 ± 0.34	1.72 ± 0.35	1.77 ± 0.39	1.78 ± 0.35
Right hearing impairment	1.42 ± 0.21	1.38 ± 0.21	1.44 ± 0.29	1.58 ± 0.38	1.68 ± 0.24	1.64 ± 0.39
*T*-values	-0.34	0.55	1.04	0.78	0.89	0.68
*P*-values	0.68	0.07	0.31	0.47	0.34	0.49


*HG.L, left Heschl’s gyrus; SMG.R, right supramarginal gyrus; SPG.L, left superior parietal gyrus; IPL.R, right inferior parietal lobule; SFG.L, left superior frontal gyrus; SFG.R, right superior frontal gyrus.*

### Intergroup Comparison of FC Between Paired ROIs

**FIGURE 2:**
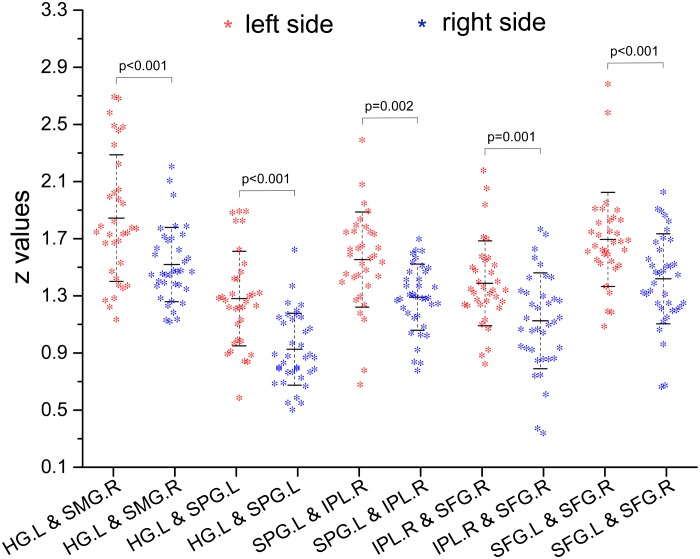
Inter-group comparison of functional connectivity (FC) (*z*-values) between paired ROIs. Compared with right hearing impairment group, the paired ROIs with enhanced FC were left Heschl’s gyrus and right supramarginal gyrus, left Heschl’s gyrus and left superior parietal gyrus, left superior parietal gyrus and right inferior parietal lobule, right inferior parietal lobule and superior frontal gyrus, and left and right superior frontal gyrus in the left hearing impairment group.

Compared with the right-sided hearing impairment group, the left-sided hearing impairment group showed enhanced FC between the left Heschl’s gyrus and right supramarginal gyrus (*P* < 0.001), left Heschl’s gyrus and left superior parietal gyrus (*P* < 0.001), left superior parietal gyrus and right inferior parietal lobule (*P* = 0.002), right inferior parietal lobule and superior frontal gyrus (*P* = 0.001), and left and right superior frontal gyri (*P* < 0.001) (Figure 2 and Table 4).

**Table 4:** Difference of inter-ROI functional connectivity between left and right hearing impairment patients groups.

	*z*-values (mean ± SD)			
			
Two brain regions	Left side	Right side	*t*-values	*P*-values
HG.L and SMG.R	1.83 ± 0.43	1.52 ± 0.26	3.84	<0.001
HG.L and SPG.L	1.29 ± 0.34	0.93 ± 0.25	4.89	<0.001
SPG.L and IPL.R	1.55 ± 0.33	1.29 ± 0.23	3.91	0.002
IPL.R and SFG.R	1.39 ± 0.29	1.13 ± 0.34	3.55	0.001
SFG.L and SFG.R	1.7 ± 0.32	1.42 ± 0.31	3.69	<0.001


*HG.L, left Heschl’s gyrus; SMG.R, right supramarginal gyrus; SPG.L, left superior parietal gyrus; IPL.R, right inferior parietal lobule; SFG.R, right superior frontal gyrus.*

### Correlation Analysis

**FIGURE 3:**
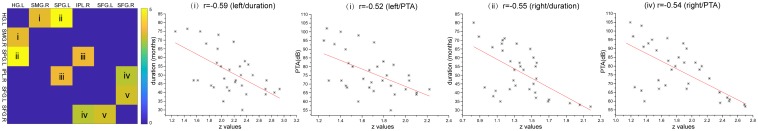
Correlations between altered FC (*z*-values) of paired regions and corresponding duration and PTA scores of the patients (altered FC showed from i to v; the colorbar indicated *t*-values among bilateral comparisons in the Table 4 which showed the degree of difference of inter-groups). In the left hearing impairment group, the FC of paired ROIs that correlated with the duration and PTA were left Heschl’s gyrus and right supramarginal gyrus (i). In the right hearing impairment group, the FC of paired ROIs that correlated with the duration were the left Heschl’s gyrus and superior parietal gyrus (ii), and correlated with the PTA were the right inferior parietal lobule and superior frontal gyrus (iv).

In the left-sided hearing impairment group, there was a significant correlation between the FC of paired ROIs and the duration of illness, as well as between the FC of paired ROIs and PTA scores in the left Heschl’s gyrus and right supramarginal gyrus (duration: *P* < 0.001, *r* = -0.59; PTA scores: *P* < 0.01, *r* = -0.52). In the right-sided hearing impairment group, there were significant correlations between the FC of paired ROIs and duration of illness in the left Heschl’s gyrus and left superior parietal gyrus (*P* < 0.01, *r* = -0.55), and between the FC of ROIs and PTA scores in the right inferior parietal lobule and right superior frontal gyrus (*P* = 0.02, *r* = -0.54; Figure 3 and Table 5).

**Table 5:** Brain regions showed correlation between left and right hearing impairment groups.

Groups	Two brain regions	Correlation with	*r*-values	*P*-values
Left hearing impairment	HG.L and SMG.R	Duration	-0.59	<0.001
	HG.L and SMG.R	PTA	-0.52	0.005
Right hearing impairment	HG.L and SPG.L	Duration	-0.55	0.002
	IPL.R and SFG.R	PTA	-0.54	0.02


*HG.L, left Heschl’s gyrus; SMG.R, right supramarginal; SPG.L, left superior parietal gyrus; IPL.R, right inferior parietal lobule; SFG.R, right superior frontal gyrus.*

## Discussion

Long-term unilateral hearing impairment can lead to abnormal changes in brain function, which can lead to a variety of network abnormalities in the brain involving sensory, conductive, and cognitive functions (Chen and Young, 2016; Shah et al., 2018). Awareness of abnormal changes in brain function and related functional integration will help to elucidate the neurological mechanisms by which impairment of brain function occurs in patients with long-term unilateral hearing impairment (Chen and Young, 2016). Such awareness would also be helpful for the identification areas of abnormal brain activity that could serve as clinical therapeutic targets. In this study, we used fALFF values and FC to study changes in resting brain function activity, as well as to compare changes between patients with left-sided long-term hearing impairment and those with right-sided long-term hearing impairment.

We found that the left-sided hearing impairment group showed significantly higher fALFF values in the left superior parietal gyrus, right inferior parietal lobule, and right superior frontal gyrus, compared with those in the right-sided hearing impairment group; moreover, the left-sided hearing impairment group showed significantly lower fALFF values in the left Heschl’s gyrus, right supramarginal gyrus, and left superior frontal gyrus, compared with those in the right-sided hearing impairment group. Heschl’s gyrus, also known as the transverse temporal gyrus, is the primary cortical structure that processes incoming auditory information (Warrier et al., 2009). A previous study has shown, using fMRI, that the transverse temporal gyrus is active during auditory processing of tone and semantic tasks (Lieu et al., 2012). Interestingly, the transverse temporal gyrus in the left hemisphere has a significantly more rapid processing rate (33 Hz), relative to that in the right hemisphere (3 Hz). This difference in processing rate is associated with the volume of the rate-related cortex in the gyrus (Zatorre and Belin, 2001).

Other brain regions that showed functional changes in patients with hearing impairments in this study have not previously been shown to play direct roles in auditory processing. The superior parietal lobule is associated with spatial orientation and receives visual and sensory inputs (Goldberg et al., 2006); the inferior parietal lobule is associated with the perception of emotions in facial stimuli and interpretation of sensory information (Peeters et al., 2009); the supramarginal gyrus forms part of the somatosensory association cortex, which interprets tactile sensory data, and is associated with the perception of space and limb location (Hartwigsen et al., 2010); the right supramarginal gyrus plays a central role in controlling empathy toward other individuals. Evidence from fMRI studies has indicated that the superior frontal gyrus is involved in self-awareness, in coordination with the actions of the sensory system (Morosan et al., 2005). The activation of the inferior parietal lobule is related to the recognition of linguistic information, while the functions of the superior frontal lobe and the middle gyrus are associated with the retention of linguistic information (Pross et al., 2015). Some studies have suggested that equivalent information about the environment can be obtained by perceptual compensation through perception of stimuli that are suitable for their own sensory patterns (Leclerc et al., 2000). Moreover, Leclerc et al. (2000) revealed that the visual area of the brain is used to perform auditory functions in individuals who are blind. Therefore, the activity observed in non-auditory areas in patients with unilateral hearing impairment in our study supports the perceptual compensation theory.

In previous studies, altered functional activity in patients with hearing impairment has been reported in the inferior frontal gyrus, superior marginal gyrus, anterior central gyrus, and posterior central gyrus (Schmithorst et al., 2014; Zhang et al., 2015). Furthermore, abnormal activation of the central anterior and posterior gyri of patients with hearing impairments is significantly higher than that in healthy controls (Bertolero et al., 2015; Chang et al., 2016; Zhang et al., 2016 Paul et al., 2017). However, in the present study, no significant differences in activation were found in the anterior and posterior gyri between patients and controls. Bushara et al. (1999) analyzed auditory and visual processing and found extensive activation in regions in the parietal lobes and inferior frontal gyrus where integration of auditory and visual information is performed. Abnormal functional activity in these regions of the brain suggests that brain reorganization might occur in patients with neurological deafness (Wang et al., 2014). Furthermore, these results indicate that the functional networks of the human brain undergo neuroplasticity under a variety of physiological conditions, in order to adapt to environmental needs and changes. Our results are inconsistent with those of previous studies. Therefore, we speculate that the mechanism of reorganization of the resting-state auditory event-related network in patients with long-term hearing impairment is complex and requires further study.

In the present study, the overall FC of each ROI was compared between the left- and right-sided hearing impairment groups. No differences were identified. The balance of overall connectivity strength in different functional regions of the brain might have been maintained because remodeling of the functional activity of the brain in these patients compensated for areas of the brain that were functionally damaged. Comparisons of FC between left- and right-sided hearing impairment groups showed that the paired regions with enhanced FC in the left-sided hearing impairment group were left Heschl’s gyrus and right supramarginal gyrus, left Heschl’s gyrus and left superior parietal gyrus, left superior parietal gyrus and right inferior parietal lobule, right inferior parietal lobule and right superior frontal gyrus, and left and right superior frontal gyrus. These results indicated that FC changes in these regions related to information input may contribute to remodeling of the sensory system. Furthermore, these results highlighted the role of Heschl’s gyrus in processing incoming auditory information (Zhang et al., 2017b). In addition, our results indicated that the FC of the regions of the brain in the resting-state network related to auditory information was stronger in the left side than in the right side, which is consistent with the results of another study (Puschmann and Thiel, 2017). The functional integration network of patients with left-sided hearing impairment underwent more obvious reorganization, indicating a laterality of processing with respect to incoming auditory information in the brain; this is consistent with the findings of previous studies (Wang et al., 2016). Furthermore, we showed an asymmetry in the structure and function of the left and right hemispheres of the brain, which is consistent with the findings of a previous study that showed lateralization in the processing of auditory sensory afferent information with a dominant hemisphere effect (Burton et al., 2012).

In patients with left-sided hearing impairment, the paired ROI that showed negative correlations with both the duration of illness and PTA scores was the left Heschl’s gyrus and right supramarginal gyrus. In patients with right-sided hearing impairment, the paired ROIs that showed respective negative correlations with duration and PTA scores were the left Heschl’s gyrus and left superior parietal gyrus, and the right inferior parietal lobule and superior frontal gyrus. The processing of auditory information is associated with sensory transmission. Therefore, we speculate that increased sensory afferent actions may accompany increased disease severity. This may explain the close relationship between disease severity and changes in the auditory information processing in patients with hearing impairment.

This study had some limitations. First, the sample size was relatively small. Therefore, the validity of the statistical inferences is relatively low. Second, the noise of high-field magnetic resonance could not be completely eliminated with the earplugs and sponge pads that were placed on the head during the experiment; this may have been more obvious for the healthy control participants. Finally, we did not differentiate among etiologies for unilateral hearing impairment. In the future, we plan to increase our sample size, use etiological grouping, and eliminate the impact of noise from magnetic resonance, in order to confirm the findings of the present study.

In summary, this R-fMRI study provided considerable evidence for abnormal intrinsic brain activity in patients with unilateral hearing impairment. Furthermore, our data indicated differences in the mechanism of reintegration between patients with left-sided hearing impairment and those with right-sided hearing impairment. Finally, we found that the disease severity was associated with differences in functional activity in certain regions of the brain. However, further studies are needed to explore the specific underlying mechanisms by which fALFF values and functional activity are altered in patients with hearing loss.

## Data Availability

The datasets generated for this study are available on request to the corresponding author.

## Author Contributions

XX, YL, and XH acquired and analyzed the data, drafted and revised the manuscript. PL, XH, and HQ analyzed and explained the data. JL and HY revised the manuscript for extremely important intellectual content.

## Conflict of Interest Statement

The authors declare that the research was conducted in the absence of any commercial or financial relationships that could be construed as a potential conflict of interest.

## Footnotes

1 https://www.fil.ion.ucl.ac.uk/spm/software/spm12/
